# The Value of Determining the Opsonic Index in the Treatment of Chronic Infections with Vaccines

**Published:** 1908-04

**Authors:** J. Edgar Paullin


### THE VALUE OF DETERMINING THE OPSONIC INDEX IN THE TREATMENT OF CHRONIC INFECTIONS WITH VACCINES.

BY J. EDGAR PAULLIN, B. A., M. D.

(*From the Laboratory of State Board of Health.*)

Recently an abundance of literature has appeared dealing with the practicability and feasibility of determining the opsonic index in reference to the administration of vaccines. There seems to be a considerable difference of opinion among most of the writers as to whether the time spent in going through the long procedure necessary to make this determination is worth the while, and as to whether it gives an accurate idea as to the exact state of the patient's blood. Perhaps it will not be amiss to briefly review some of the objections to this determination, and, at the same time, point out the fact that therapeutic vaccines can be, and are, administered without the tedious procedure of determining the opsonic index.

In a previous resume on opsonins (Medical Consensus, February, 1907) it was attempted to briefly outline the technique which was employed in the estimation of the index. In order to understand the sources of error, it perhaps is best to mention briefly the procedures ordinarily employed. As stated then, three solutions are necessary:

(1.) An even bacterial suspension in normal salt solution free from clumps.

(2.) Washed white blood corpuscles.

(3.) Serum to be tested.

Regarding the white blood corpuscles, it is generally advisable that they should be corpuscles from the patient whose index is to be determined. We next prepare what Wright has fitly called a "pool of serum"—meaning by this, serum from four or five normal individuals. This is used for the purpose of comparing with the serum to be tested. Preparations are then made containing equal parts of serum, bacterial suspension and washed corpuscles. These are incubated at 37 degrees Centigrade fifteen minutes. Smears are then made and stained with some of the aniline dyes,—preferably Hasting's solution. One then counts the number of bacteria contained in a certain number,—usually 50 polymorpho-nuclear white cells. In examining such a preparation one observes that not all of the white blood cells contain bacteria, so that we generally take into account the number of cells containing bacteria and those not containing bacteria. Comparing these results we observe what is called the phagocytic index.

The result obtained from the "pool of serum" is taken to be normal, while that obtained from the serum to be tested is compared with the "pool" and is considered to be either above or below normal, according to whether the leukocytes contain a greater or less number of bacteria than the supposedly normal one.

To one who has had experience with this particular technique, the sources of error are quite numerous. In the first place, perhaps the greatest source of error lies in what is called the "personal equation,"—a factor which cannot be eliminated from any of the determinations. This is very well illustrated by the accompanying table, which is a series of three counts made on the same slide by three individuals who had had considerable experience in this work. The bacteria were counted in 50, 100 and 150 leukocytes respectively, and the results recorded.

| | Bacteria Within 50 P W.B.C. | Bacteria Within 100 P.W.B.C. | Bacteria Within 150 P.W B-C. | Lowest Bacteria Per Cell | Highest Bacteria Per Cell | Difference Between Highest and Lowest |
|---|---|---|---|---|---|---|
| A's Counts | 76 | 165 | 235 | ------ | ------ | ------- |
| A's Bacteria Per Cell | 1.5 | 1.65 | 1,56* | 1.5 | 1.65 | 0.15 |
| B's Counts | 87 | 1[illegible]0 | 268 | ---- | ------ | ------- |
| B's Bacteria Per Cell | 1.74 | 1.6 | 1.78 | 1 6 | 1.78 | 0.18 |
| C's Count | 80 | 169 | 252 | ------ | ------ | ------- |
| C's Bacteria Per Cell | 1-8 | 1.69 | 1.68 | 1.68 | 1.8 | 0.12 |

From this table we observe that there is a considerable difference between the three counts. The highest number of bacteria in 50 P. W. B. C. is 87, whereas the lowest is 76 P. W. B. C. In the count of 150 P. W. B. C. the highest count is 268, while the lowest is 235. It is seen from this that a considerable difference of opinion exists in the minds of workers as to whether the given organism is within or upon the given leukocyte. However, if we take the three counts of the respective individuals and compare these counts with each other, we observe that the difference between the counts in 50, 100 and 150 cells is within the limit of error, and that the number of bacteria, as figured per cell, is practically the same in the three counts, so that it is necessary as a rule in determining the index to have all counts made by the same individual.

It is generally supposed that an error of from 4 to 8 per cent. even with an individual worker is to be expected, so that from the above table we see that the individual counts come within the limit of this source of error.

As recently stated by Park and Biggs (Journal of Medical Research, Vol. 17, No. 1, Page 77), the opsonic index is by no means a fixed quantity, even for a normal individual, and they have expressed the opinion which we have long held and several times demonstrated, that there is a fluctuation in the index of normal individuals varying with the time of day and condition of the individual, whether fatigued or at rest. This was shown to be true with reference to the staphylococcus aureus, and it might readily be supposed that such a condition is true relative to other organisms. As yet, however, not enough proof is forthcoming to establish this as a fact. Since this is supposedly true for the normal individual, it might well be a question if the same fluctuation does not take place in the serum of a patient the subject of a chronic infection. In fact, it would seem reasonable that these fluctuations would be more marked under these conditions.

The authors above referred to also point out the fact that we never know the opsonic index at the time of the administration of the vaccine. The reasons for this are very briefly stated as follows: In the first place it is generally from three to twenty-four hours after obtaining the serum that we are able to arrive at a conclusion as to the index of the patient. This condition is true on account of the fact that it requires considerable time to estimate the index, and very frequently it is impossible to continue straight through the procedure after it is once commenced; since this lapse of time is necessary between the obtaining of the blood and the conclusion reached as to whether the dose of vaccine is necessary or not, supposing that it is necessary, the vaccine is administered on an index determined the day previous, and it by no means signifies that the index of the patient is the same at the time the vaccine is administered as it was when determined.

In the table, No. 2, which follows below, a series of indices were determined on three different patients, the subjects of chronic infections.

| Date | 19 | 20 | 21 | 22 | 23 | 24 | 25 | 26 | 27 |
|---|---|---|---|---|---|---|---|---|---|
| Patient A., Opsonic Index...... | I.2 | 1.0 | 1.3 | 1.0 | 0.9 | 0.9 | 1.1 | 1.3 | 0.9 |
| Patient B., Opsonic Index ...... | 0.6 | 0.7 | 0.6 | 1.0 | 1.1 | 0.7 | 0.8 | 0.8 | 0.6 |
| Patient C., Opsonic Index ...... | 0-9 | 0.8 | 0.7 | 0.7 | 0.6 | 0.7 | 0.6 | 0.7 | 0.7 |

From this table one notices that the opsonic index varies greatly in the same patient from day to day; these determinations were made before the administration of the vaccine in order to find out, if possible, whether the patient's condition warranted vaccine therapy. In two of the cases one observes that the index was persistently below the normal. However, it is noticeable that in one, patient B., the index on one occasion was normal and on the other above normal. It is perfectly conceivable that had these indices been determined only on these two occasions that those believing implicitly in the value of determining the index would not have administered vaccine, whereas, clinically, it was observed that vaccine therapy was clearly indicated.

From the foregoing facts we have in our work practically abandoned the determination of the index, and we are convinced quite firmly that by observing the clinical condition of the patient we are often able to determine satisfactorily as to whether the administration of the vaccine is necessary or not.

Our experience with vaccine therapy now amounts to something like fifty cases, in which we have administered vaccines without determining the index. These cases are those of tubercular lymph glands, tuberculosis of the bone, tuberculosis of the skin, furunculosis, chronic empyema, chronic pustular acne and chronic gonorrhœal arthritis. In the greater number of these cases improvement has been quite marked, and we feel justified in stating that the administration of vaccines can be carried out as satisfactorily without the determination of the opsonic index.

